# Glycyrrhizin improves the pathogenesis of psoriasis partially through IL-17A and the SIRT1-STAT3 axis

**DOI:** 10.1186/s12865-021-00421-z

**Published:** 2021-05-27

**Authors:** Huang Qiong, Ling Han, Nanxue Zhang, Huyan Chen, Kexiang Yan, Zhenghua Zhang, Ying Ma, Jinhua Xu

**Affiliations:** Department of Dermatology, Huashan Hospital Affiliated to Fudan University, Shanghai, 200040 China

**Keywords:** Psoriasis, Glycyrrhizin, IL-17A, SIRT1-STAT3 pathway

## Abstract

**Background:**

The anti-inflammatory effect of glycyrrhizin has been widely recognized, while the specific mechanism of glycyrrhizin in psoriasis remains poorly understood.

**Results:**

In the imiquimod-induced mouse model of psoriasis (IMD), we found that glycyrrhizin can substantially improve the adverse symptoms in mice. The hematoxylin-eosin staining results showed that glycyrrhizin can also improve the pathological state of skin cells in IMD mice. Using enzyme-linked immunosorbent assay (ELISA), we found that glycyrrhizin substantially inhibited the expression of IL-17A and IFN-γ in the serum of IMD mice. In order to simulate the effect of IL-17A on keratinocytes in psoriasis, we treated HaCaT cells with 100 ng/mL IL-17A (IL-17A-HaCaT cells) for 48 h. Then, using cell-counting kit-8 (CCK-8) and ELISA assays, we found that glycyrrhizin inhibited the proliferation of IL-17A-HaCaT cells and reversed the promotion of IL-6, CCL20, and TNF-α induced by IL-17A. Further, western blotting (WB) results indicated that glycyrrhizin promoted the expression of SIRT1 and inhibited the expression of STAT3 and phosphorylated STAT3 (p-STAT3). By treating IL-17A-HaCaT cells with EX-527 (a potent and selective inhibitor of SIRT1), combined with CCK-8 and WB experiments, we initially found that EX-527 inhibited the proliferation of IL-17A-HaCaT cells and promoted the expression of STAT3, p-STAT3, and acetylated STAT3 (a-STAT3). However, when glycyrrhizin was added at the same time, the proliferation of IL-17A-HaCaT cells increased, and the expression of STAT3, p-STAT3, and a-STAT3 reduced. We then knocked down the expression of SIRT1 via small interfering RNA in IL-17A-HaCaT cells, and the results were consistent with those of EX-527.

**Conclusions:**

Together, these results indicated that glycyrrhizin improved psoriasis by inhibiting the expression of IL-17A and IFN-γ in vivo and suppressed the proliferation of IL-17A-HaCaT cells and the expression of STAT3, p-STAT3, and a-STAT3 by upregulating SIRT1 in vitro.

**Supplementary Information:**

The online version contains supplementary material available at 10.1186/s12865-021-00421-z.

## Highlight

1. Glycyrrhizin improves psoriasis by reducing the secretion of IL-17A and IFN-γ in serum.
2. Glycyrrhizin may inhibit the proliferation of keratinocytes by reducing the expression of IL-17A and IFN-γ.
3. Glycyrrhizin may inhibit the proliferation of keratinocytes through the SIRT1-STAT3 pathway.
4. Glycyrrhizin inhibits the expression of p-STAT3 and a-STAT3 through a SIRT1-dependent pathway

## Introduction

Clinically, glycyrrhizin is often used to treat patients with acute and chronic hepatitis, liver poisoning, and early liver cirrhosis [1, 2], and the efficacy of glycyrrhizin in improving psoriasis has attracted the attention of clinicians. A previous study showed that glycyrrhizin combined with methotrexate (MTX) can be used as an effective alternative therapy for the treatment of erythrodermic psoriasis with bullous pemphigoid [3]. Moreover, glycyrrhizin combined with acitretin can improve psoriasis by regulating T helper 17 (TH17) cells [3]. However, the clinical value of glycyrrhizin in psoriasis has not been fully developed.

The imbalance in the dynamic interaction of immune cells and keratinocytes plays important roles in the initial and maintenance stages of psoriasis [4]. Psoriasis is considered as the chronic T helper 1(TH1)/TH17-mediated inflammatory disease [5]. IL-17A is mainly synthesized and secreted by TH17 cells [6] and is considered to play a key role in the pathogenesis of psoriasis [7, 8]. Moreover, IL-17A is also key to the interaction between TH17 cells and keratinocytes, specifically, the secretion of many cytokines and chemokines, such as TNF-α, IL-6, IL-17A and CCL20 in keratinocytes were stimulated by TH17 cells-secreted IL-17A [9, 10]. Therefore, we aimed to further explore the relationships between glycyrrhizin, TH17 cells, and keratinocytes.

The imbalance between pro-inflammatory and anti-inflammatory signals may lead to the development of psoriasis [11]. Keratinocytes are the main source of inhibitory cytokines, which maintains the skin in an inflammatory quiescent state [4]. A previous study has shown that SIRT1 inhibits keratinocyte proliferation and promotes keratinocyte differentiation [12]. Therefore, SIRT1 may regulate the release of inhibitory cytokines by affecting the keratinocyte proliferation and play a key role in the occurrence and development of psoriasis.

A previous study reported that oxidative stress contributes to the pathogenesis of psoriasis, and the activation of SIRT1 inhibits the MAPK, NF-κB, and STAT3 oxidative stress signaling pathways, thereby down-regulating inflammatory factors and inhibiting excessive keratinocyte proliferation [13]. The activation of STAT3 in keratinocytes partly stimulates the activation of Langerhans cells through IL-1α, and their presence is vital to the pathogenesis of psoriasis through the production of IL-23 [11]. Therefore, we speculate that the SIRT1-STAT3 axis may be a potential mechanism for glycyrrhizin to regulate the pathogenesis of psoriasis, which is worthy of further study.

Based on the above findings, our study sheds light on the following important connections between glycyrrhizin, IL-17A, SIRT1, STAT3, and the pathogenesis of psoriasis. First, we set out to characterize the regulatory relationship between glycyrrhizin and IL-17A in vivo. We found that glycyrrhizin inhibits the expression of IL-17A and its related genes, *IL-6, CCL20*, and *TNF-α*. In addition, the regulatory relationship between glycyrrhizin and the SIRT1-STAT3 axis was investigated in vitro*,* and the results showed that glycyrrhizin inhibits STAT3 expression by promoting SIRT1 expression. In summary, these data indicate that glycyrrhizin may play a therapeutic role in improving psoriasis through IL-17A and the SIRT1-STAT3 axis.

## Materials and methods

### Clinical samples

Clinical skin samples from female patients with psoriasis and healthy persons (mean age: psoriasis 38.6 years vs. healthy volunteers 41.3 years) were collected from Huashan Hospital (Shanghai, China). Under local anesthesia, 4 mm punch biopsy samples were obtained from the lower limb skin of psoriasis patients and healthy donors. The experiment was approved by the Huashan Hospital Clinical Research Ethics Committee. All methods were carried out in accordance with relevant guidelines and regulations, and all participants/donors provided written informed consent for the study.

### Animal experiments

All male BALB/c mice (10 weeks old, 25–35 g) were obtained from the Animal Experiment Centre of Huashan Hospital and fed under sterile specific-pathogen-free (SPF) conditions. The mice were randomly assigned into four groups (*n* = 6 each) for further study, and the mice in group A were not treated. In group B-D, mice were evenly smeared with 62.5 mg imiquimod cream on their backs once a day for three consecutive days. On the fourth day, 50 mg imiquimod cream was applied evenly on the backs of the mice once a day for two consecutive days. On day seven, the psoriasis animal models (imiquimod-induced mouse model of psoriasis, IMD) were successfully established in the B-D groups. In group B, normal saline was intragastrical injected for the positive control group (model group). MTX has been used to treat psoriasis and other skin diseases for more than 50 years [13]. For mice in group C, 20 mg/kg MTX was intragastrical injected for the treatment control group (model + MTX group). In addition, in mice of group D, 20 mg/kg glycyrrhizin was intragastrical injected for the treatment observation group (model + glycyrrhizin group). In groups B–D, the treatment was performed once a day for four consecutive days, and on the eleventh day, the back skin of the mice in groups A-D was photographed, and the mice were sacrificed by cervical dislocation. On the eleventh day, the Psoriasis Area and Severity Index (PASI) was used to monitor and grade the severity of psoriasis-like lesions. The detection indicators included skin erythema, scale and thickness. Among them, 0 represents no clinical signs; 1 represents mild clinical signs; 2 represents moderate clinical signs; 3 represents obvious clinical signs; 4 represents very obvious clinical signs [14]. The animal experiments used meet the requirements of animal welfare and animal ethics of Huashan Hospital, and are approved by the Ethics Committee of animal experiments of Huashan Hospital. All animal experiments met the ARRIVE guidelines [15], and the mice were anesthetized with isoflurane and then sacrificed by cervical dislocation. The experimental design in vivo was shown in supplement Figure 1.

### HE staining

After the tissues were baked, dewaxed and rehydrated, they were stained with hematoxylin for 10 min and eosin for 8 min, and were treated with xylene I, xylene II, and xylene III for transparency. The infiltration of immune cells was evaluated by two pathologists in a double-blind manner.

### Cell culture and treatment

HaCaT cells were purchased from the cell bank of the Chinese Academy of Sciences and met the cell line STR identification criteria. HaCaT cells were cultured in Dulbecco’s Modified Eagle’s Medium (L110, Basamedia Biology, Shanghai, China) supplemented with 10% fetal bovine serum (Thermo Fisher), 100 g/mL penicillin, and 100 g/mL streptomycin (L110, Basamedia Biology, Shanghai, China) in a moist incubator with 5% CO2 at 37 °C. Wild type HaCaT cells were treated with IL-17A (100 ng/mL), glycyrrhizin (0.5, 1.0, 1.5 and 2 μM), and IL-17A (100 ng/mL) + glycyrrhizin (2 μM) for 48 h. The IL-17A-HaCaT cells (treated with 100 ng/mL IL-17A) were treated with glycyrrhizin (2 μM) and EX527 (100 nM, SIRT1 inhibitor, Med Chem Express) for 48 h.

### Cell-counting kit-8 (CCK-8)

HaCaT cells were seeded in a 96-well plate at a density of 5 × 10^3^ cells per well (triple replication). The next day, after the HaCaT cells adhered, the culture solution was discarded, and a mixture of 10 μL CCK-8 solution + 90 μL serum-free medium was added, and the cells were cultured in an incubator for 2 h. After 2 h, the absorbance of the cells at 450 nm was measured using a microplate reader (SpectraMax M2e; Molecular Devices, Sunnyvale, CA, USA). The above steps were repeated for five consecutive days.

### Immunohistochemical experiments

For the immunohistochemical analyses, skin samples were dewaxed and rehydrated, followed by endogenous peroxidase quenching, antigen retrieval (saline sodium citrate, autoclaving), and blocking with 10% goat serum. The sections were then incubated with anti-rabbit STAT3 antibody (1:2000, CST, USA), phosphorylated STAT3 (p-STAT3) antibody (1:2000, CST, USA), acetylated STAT3 (a-STAT3) antibody (1:2000, CST, USA), SIRT1 antibody (1:2000, CST, USA), AMPK antibody (1:2000, CST, USA), phosphorylated AMPK (p-AMPK) antibody (1:2000, CST, USA), and GAPDH antibody (1:2000, CST, USA). After washing with PBS, the sections were incubated for 50 min with secondary antibody, stained with DAB working solution, and then counterstained with hematoxylin.

### Enzyme linked immunosorbent assay (ELISA)

The cell lysis solution was collected to detect the expression of IL-6 (1:2000, Abcam, USA), TNF-α (1:2000, Abcam, USA), and CCL20 (1:2000, Abcam, USA) according to the manufacturer’ s instructions. The absorbance was then measured with a microplate reader (SpectraMax M2e, USA) at 450 nm.

### Real-time fluorescence quantitative PCR (RT-PCR)

Trizol reagent (Thermo Fisher Scientific) was used to extract total RNA from cells and tissues, and a reverse transcription kit (TaKaRa) was used to reverse the total RNA to cDNA. The primers used in this experiment were listed in Table 1. The fluorescent quantitative PCR kit (TaKaRa) was used to perform RT-PCR and 2-ΔΔCT was used to calculate the relative gene expression. GAPDH was used as an internal reference.

**Table 1:** The primers used in this experiment

Gene	Forward Primer (5′ - > 3′)	Reverse Primer
Human STAT3	CAGCAGCTTGACACACGGTA	AAACACCAAAGTGGCATGTGA
Mouse STAT3	CACCTTGGATTGAGAGTCAAGAC	AGGAATCGGCTATATTGCTGGT
Human SIRT1	TAGCCTTGTCAGATAAGGAAGGA	ACAGCTTCACAGTCAACTTTGT
Mouse SIRT1	TGATTGGCACCGATCCTCG	CCACAGCGTCATATCATCCAG
Human GAPDH	ACCTTAACCGCCTTATTAGCCA	CACCACGGTACAACAGGCA
Mouse GAPDH	CCTAAACAGGTTGATAGGCCAAA	CTCGCCTTCCACAGAATCCA

### Western blotting

The protein concentrations were determined using the bicinchoninic acid protein assay kit (Beyotime, China). All protein concentrations were adjusted to 2 mg/mL, and then separated by 8% SDS-PAGE. Then, the proteins were transferred to a nitrocellulose membrane (731,809, Millipore, USA) and blocked with 5% skimmed milk for 1 h, and the membrane was sequentially incubated with a primary antibody and a horseradish peroxidase-conjugated secondary antibody. The antibodies used in WB experiments were the same as those used in immunohistochemistry experiments. Final, the enhanced chemiluminescence method was used to detect protein levels.

### Cell transfection

HaCaT cells in the logarithmic phase were incubated in six-well plates at a density of 5 × 10^4^ cells/mL overnight. The next day, HaCaT cells were transfected with siRNAs (Table 2) using Lipofectamine 3000 (L3000015, Thermo Fisher Scientific), and incubated for 24 h in accordance with the manufacturer’s instructions. Then, fresh media was added, and the corresponding stimulant was added to the media for 48 h.

**Table 2:** Sequences of the siRNA oligonucleotides

Name	Sense (5′-3′)	Antisense (5′-3′)
siRNA1	CCAUCUCUCUGUCACAAAUTT	AUUUGUGACAGAGAGAUGGTT
siRNA2	CCCUGUAAAGCUUUCAGAATT	UUCUGAAAGCUUUACAGGGTT
siRNA3	GGAGAUGAUCAAGAGGCAATT	UUGCCUCUUGAUCAUCUCCTT

### Statistical analysis

Data are presented as the mean ± S.D. of at least three independent experiments. Non-parametric statistical tests of variance was performed using SPSS 19.0 software (SPSS Inc., Chicago, IL, USA) to determine the statistical significance of the results between two groups. *P* < 0.05 was considered statistically significant.

## Results

### Glycyrrhizin can improve the adverse symptoms of psoriasis in IMD

**Fig. 1:**
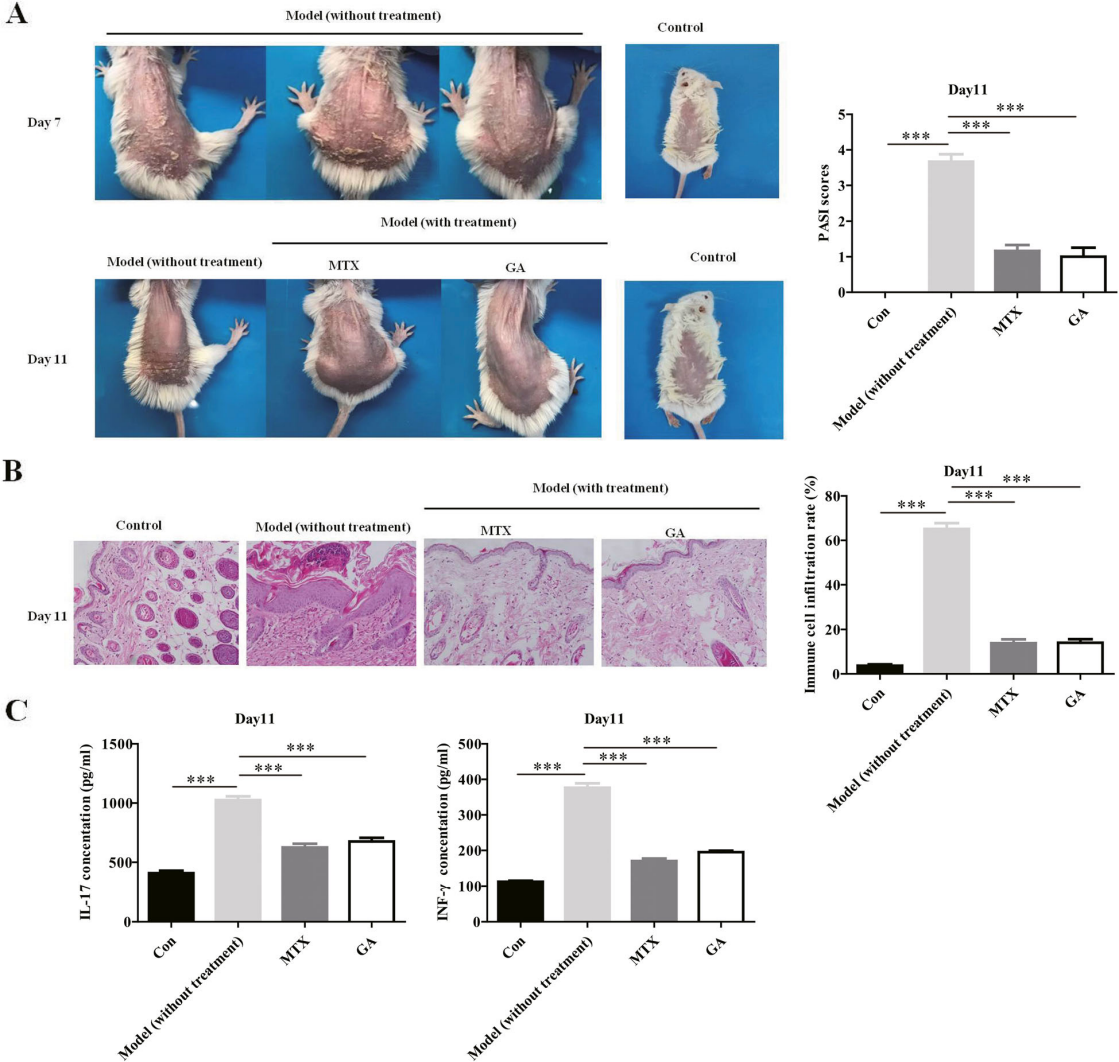
Glycyrrhizin may improve the adverse symptoms and pathological status of IMD by inhibiting the expression of IL-17A and IFN-γ. **a** Compared with the control IMD mice (treated with normal saline), after continuous treatment with 20 mg/kg MTX or 20 mg/kg glycyrrhizin for 4 days, the adverse symptoms of IMD were substantially improved by MTX or glycyrrhizin. **b** The HE staining experiments show that MTX or glycyrrhizin substantially improved the pathological status of the skin cells in IMD. **c** By the ELISA experiments, we found that the pro-inflammatory cytokines IL-17A and INF-γ were substantially inhibited by MTX or glycyrrhizin in the serum of IMD. NOTES: Data are presented as the mean ± SD (*n* = 3). ***P < 0.001; GA represents glycyrrhizin; IL-17 represents IL-17A; Model represents IMD mice

As shown in Fig. 1a (upper part), on the seventh day, the obvious clinical symptoms of psoriatic lesions appeared on the back skin of IMD mice, specifically as keratinocyte hyperproliferation, erythema, scales, etc. However, after four consecutive days of treatment with MTX or glycyrrhizin, the adverse symptoms of IMD mice substantially improved (Fig. 1a, lower part). Furthermore, on the 11th day, HE staining showed cytokeratinization and infiltration of inflammatory cells in the skin cells of untreated IMD mice, while these pathological phenomena substantially improved in the skin cells of MTX- or glycyrrhizin-treated IMD mice (Fig. 1b). In order to further investigate whether the potential mechanism of glycyrrhizin for the treatment of psoriasis involves the regulation of the inflammatory response, ELISA was used to detect the expression of IL-17A and IFN-γ in the serum of wild-type mice or IMD mice, with or without treatment. The results showed that IL-17A and IFN-γ expression in the serum of IMD mice treated with MTX or glycyrrhizin was substantially reduced compared to expression levels in non-treated mice (Fig. 1c). Together, glycyrrhizin may improve the clinical symptoms and pathological status of IMD by inhibiting the expression of IL-17A and IFN-γ in the serum.

### Glyrrhizin inhibits the expression of IL-17A-related inflammatory cytokines in HaCaT cells through its cytotoxicity

**Fig. 2:**
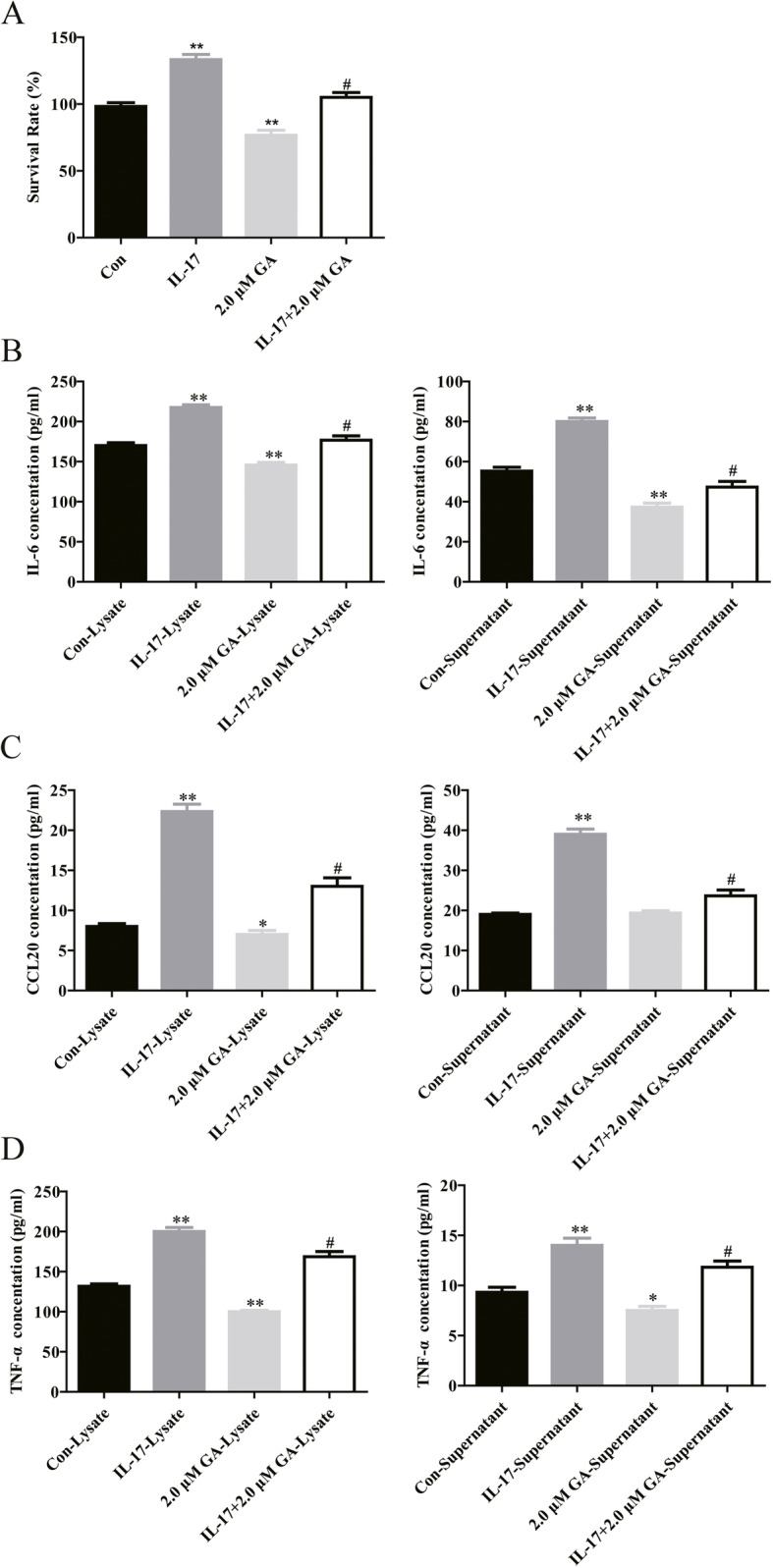
Glycyrrhizin suppresses cell proliferation and the expression of inflammatory factors in HaCaT cells. **a** The CCK-8 results indicate that IL-17A promoted the proliferation of HaCaT cells, while glycyrrhizin inhibited the proliferation of HaCaT cells. **b**-**d** After treatment with IL-17A for 48 h, the expression of IL-6 was substantially higher in the lysate and supernatant of HaCaT cells, while glycyrrhizin could reverse the promotion of IL-6 induced by IL-17A (**b**). Besides, glycyrrhizin has the same regulation of CCL20 (**c**) and TNF-α (**d**) as IL-6. NOTES: Data are presented as mean ± SD (*n* = 3). *P < 0.05, **P < 0.01 and ***P < 0.001 vs control; Con represents wild type HaCaT cells; GA represents glycyrrhizin; IL-17 represents IL-17A; #*P* < 0.05 vs IL-17A lysate or supernatant group

As shown in Supplement Figure 2 A, glycyrrhizin inhibits the proliferation of HaCaT cells in a dose-dependent manner, and 1.0–2.0 μM glycyrrhizin are cytotoxic. In addition, only 2 μM glycyrrhizin can significantly inhibit the secretion of IL-6 (Supplement Figure 2 B), CCL-20 (Supplement Figure 2 C) and TNF-α (Supplement Figure 2 D) in HaCaT cells. Therefore, glycyrrhizin-mediated inflammation inhibition is due to its own cytotoxicity. The CCK-8 results showed that 2 μM glycyrrhizin substantially reduced the proliferation of HaCaT cells, while IL-17A substantially promoted the proliferation of HaCaT cells (Fig. 2a). Compared with IL-17A group, the proliferation of HaCaT cells was substantially reduced in IL-17A + 2 μM glycyrrhizin group (Fig. 2a). In addition, as shown in Fig. 2b, the expression of IL-6 and CCL20 substantially increased in the lysate and supernatant of IL-17A-HaCaT cells. However, when glycyrrhizin was added, glycyrrhizin substantially reversed the increased expression of IL-6 (Fig. 2b) and CCL20 (Fig. 2c) induced by IL-17A. Moreover, the ELISA results showed that glycyrrhizin substantially reduced the expression of TNF-α in the lysate and supernatant of HaCaT cells and effectively reversed TNF-α stimulation by IL-17A (Fig. 2d). Together, glycyrrhizin suppresses the expression of inflammatory cytokines related to IL-17A in HaCaT cells due to its own cytotoxicity.

### Glycyrrhizin promotes SIRT1 expression and reduces STAT3 expression

**Fig. 3:**
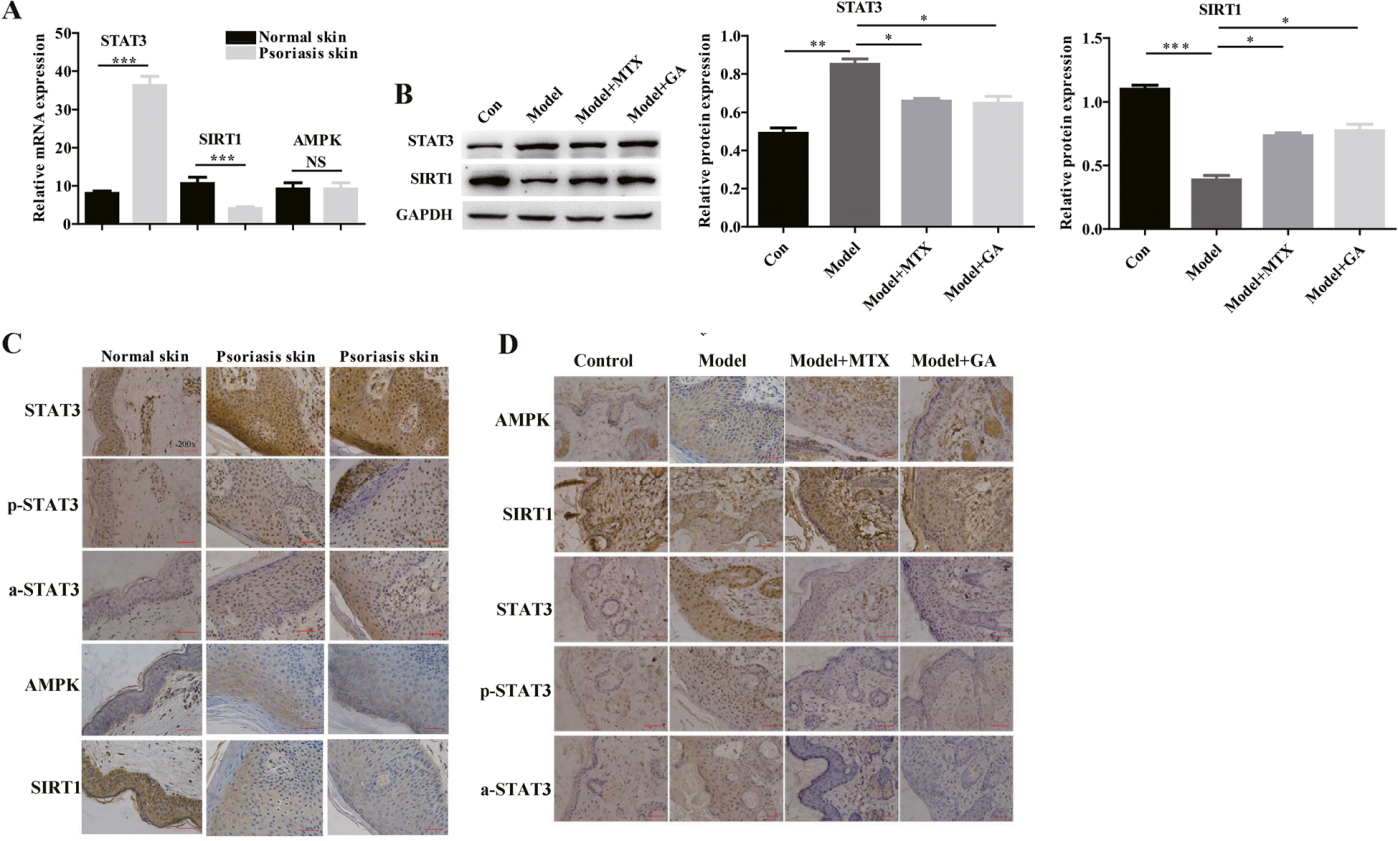
Glycyrrhizin regulates SIRT1 and STAT3 expression in vivo. **a** RT-PCR results showed that the mRNA expression of STAT3 was up-regulated while SIRT1 was down-regulated in human psoriasis skin tissues (*N* = 10) than normal skin tissues (*N* = 10). **b** western blotting results showed that compared with the skin tissues of IMD model, the protein expression of STAT3 was down-regulated while SIRT1 was up-regulated in Model+MTX or Model+GA group. **c** The immunohistochemical results showed that compared with normal subjects, the expression of STAT3 were substantially increased, and the expression of SIRT1 was substantially decreased, while the expression of AMPK was not substantially changed. **d** In IMD, the immunohistochemical results showed that when treated with glycyrrhizin, the expression of STAT3 was decreased while the expression of SIRT1 was increased in IMD. *P < 0.05, **P < 0.01 and ***P < 0.001

**Fig. 4:**
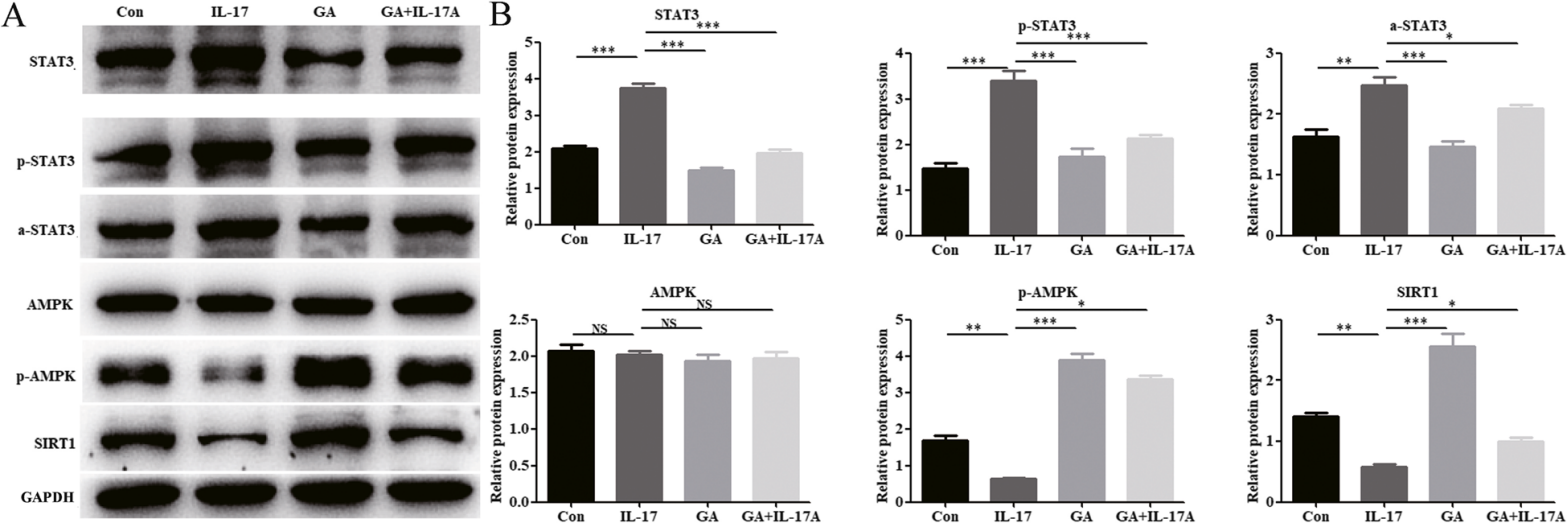
Glycyrrhizin regulates the expression of SIRT1 and STAT3 in IL-17A-HaCaT cells. **a** In IL-17A-HaCaT cells, glycyrrhizin can substantially reverse the high expression of STAT3 and p-STAT3 while reverse the low expression of SIRT1 induced by IL-17A. **b** Histogram of western blotting results. NOTES: Data are presented as the mean ± SD (*n* = 3). *P < 0.05, **P < 0.01 and ***P < 0.001; Con represents wild type HaCaT cells; GA represents glycyrrhizin; IL-17 represents IL-17A

The mRNA expression of STAT3 was up-regulated while SIRT1 was down-regulated in human psoriasis skin tissues than normal skin tissues via RT-PCR detection (Fig. 3a). However, the mRNA expression of AMPK was not statistically different between the two groups (Fig. 3a). Compared with the skin tissues of IMD model, the protein expression of STAT3 was down-regulated while SIRT1 was up-regulated in Model+MTX or Model+GA group via western blotting detection (Fig. 3b). The immunohistochemistry results showed that compared with the skin cells of normal subjects, the skin cells of patients with psoriasis showed substantially increased STAT3 expression and substantially decreased SIRT1 expression (Fig. 3c). However, the expression of AMPK, a-STAT3 and p-STAT3 were not statistically different between the two groups (Fig. 3c). The expression of STAT3, a-STAT3 and p-STAT3 were decreased and the expression of SIRT1 was increased when treated with glycyrrhizin (Fig. 3d). However, glycyrrhizin had no effect on the expression of AMPK (Fig. 3d). Moreover, glycyrrhizin substantially reversed the high expression of STAT3 and p-STAT3 and reversed the low expression of SIRT1 induced by IL-17A, at the protein level, in HaCaT cells (Fig. 4a and b). Together, these results indicate that glycyrrhizin may improve psoriasis by promoting SIRT1 expression and inhibiting STAT3 expression.

### Glycyrrhizin suppresses keratinocyte proliferation and STAT3 expression by upregulating SIRT1

**Fig. 5:**
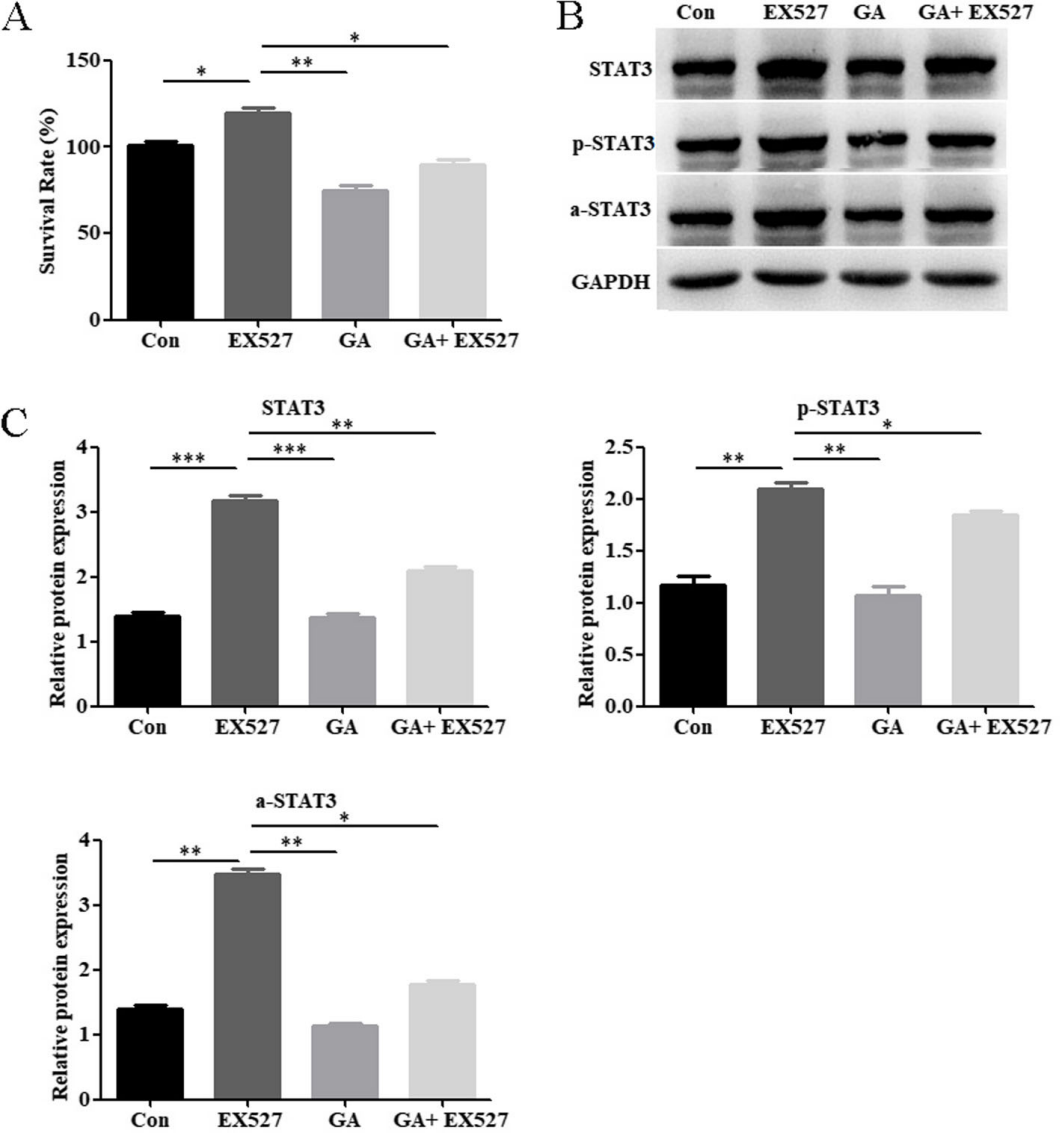
Glycyrrhizin suppresses the expression of STAT3 via SIRT1 in IL-17A-HaCaT cells. **a** The CCK-8 assay was used to determine the effect of EX527 and glycyrrhizin on proliferation of IL-17A-HaCaT cells. **b** The western blotting results show that EX527 promotes the expression of STAT3, p-STAT3, a-STAT3 in IL-17A-HaCaT cells, and glycyrrhizin can reverse the promotion of STAT3, p-STAT3 and a-STAT3 induced by EX527. **c** Histogram of western blotting statistical results. NOTES: Data are presented as the mean ± SD (*n* = 3). *P < 0.05, **P < 0.01 and ***P < 0.001; Con represents wild type HaCaT cells; GA represents glycyrrhizin; GAPDH is used for internal reference

**Fig. 6:**
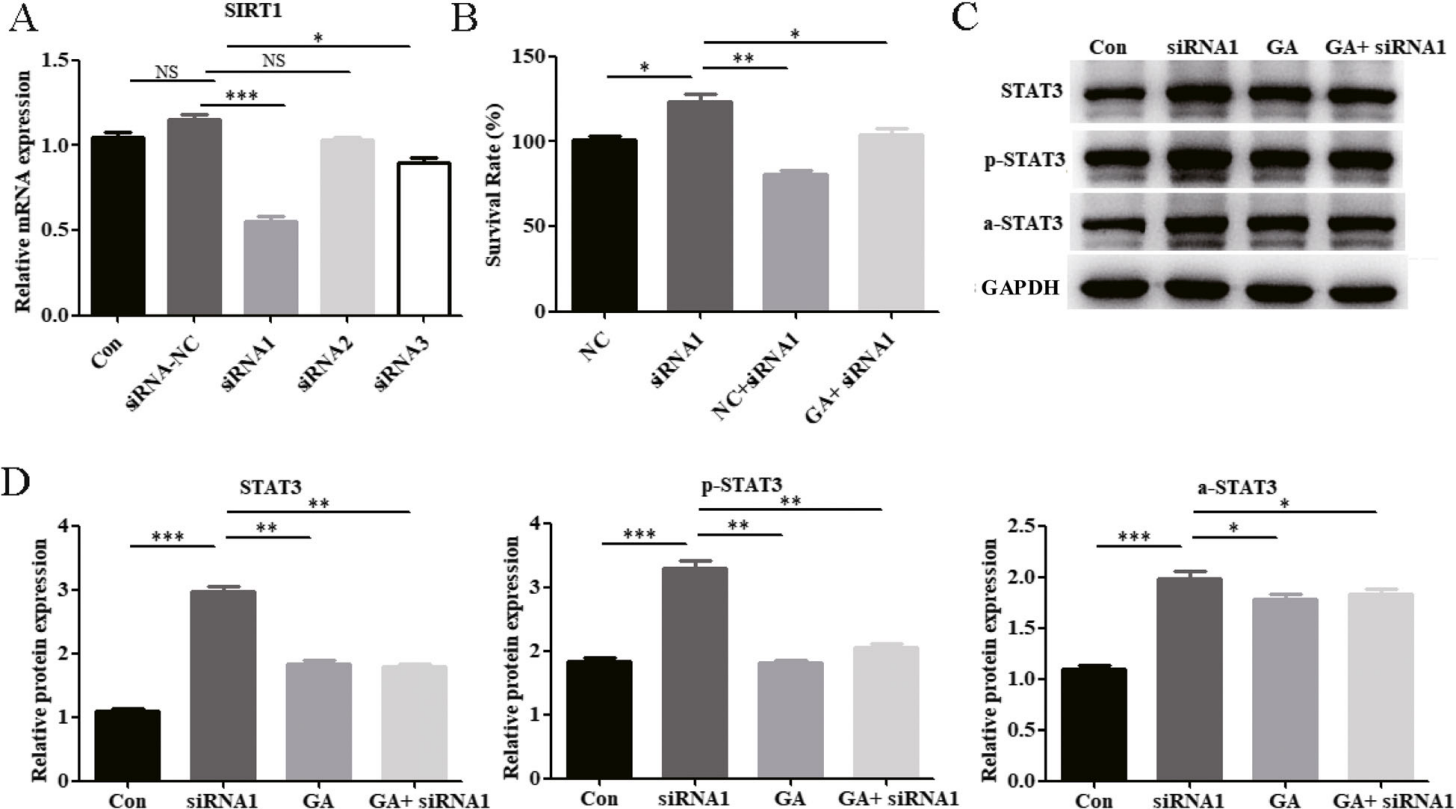
Glycyrrhizin inhibits the expression of STAT3 by up-regulating SIRT1 in IL-17A-HaCaT cells. **a** and **b** By constructing SIRT1-knockdown small interfering RNA (**a**) and combining with CCK-8 experiments (**b**), we found that SIRT1-knockdown promoted the proliferation of IL-17- HaCaT cells. **c** When knock-down SIRT1 in IL-17- HaCaT cells, glycyrrhizin could reverse the promotion of STAT3, p-STAT3 and a-STAT3 induced by SIRT1 knock-down. **d** Histogram of western blotting statistical results. NOTES: Data are presented as the mean ± SD (*n* = 3). *P < 0.05, **P < 0.01 and ***P < 0.001; NC represents siRNA-NC transfected HaCaT cells; GA represents glycyrrhizin; GAPDH is used for internal reference

As shown in Fig. 5a, EX-527 promoted the proliferation of IL-17A-HaCaT cells, while glycyrrhizin substantially reduced this effect. This finding suggests that glycyrrhizin partially reduces the proliferation of IL-17a-HaCaT cells by promoting SITR1 expression. Further, western blotting experiments showed that EX-527 substantially promoted the expression of STAT3, p-STAT3, and a-STAT3 in IL-17A-HaCaT cells. Compared with EX-527 group, the expression of STAT3, p-STAT3, and a-STAT3 were substantially reduced in glycyrrhizin+EX-527 group (Fig. 5b and c). Moreover, interfering RNAs (siRNA-1, siRNA-2, and siRNA-3) that knock down SIRT1 were constructed, and the RT-PCR results showed that siRNA-1 substantially knocked down SIRT1 expression in IL-17A-HaCaT cells (Fig. 6a). As shown in Fig. 6b, the CCK-8 results further confirmed that reduced SIRT1 expression promoted the proliferation of IL-17A-HaCaT cells. Compared with siRNA1 group, the proliferation of IL-17A-HaCaT cells were substantially reduced in glycyrrhizin+siRNA1 group (Fig. 6b). In addition, the western blotting results indicated that when IL-17A-HaCaT were treated with glycyrrhizin, the STAT3, p-STAT3, and a-STAT3 expression levels that increased upon SIRT1 knockdown were substantially reversed (Fig. 6c and d). Together, these results suggest that glycyrrhizin might partially inhibit the proliferation of keratinocytes via SIRT1-STAT3 axis, thereby playing a therapeutic role in psoriasis.

## Discussion

In this study, we characterized the biological role of glycyrrhizin in psoriasis in vivo and in vitro. Glycyrrhizin is a therapeutic substance that improves psoriasis by down-regulating IL-17A and INF-γ, and further inhibits the expression of IL-6, TNF-α, and CCL20 induced by IL-17A. Mechanically, glycyrrhizin inhibits the expression of STAT3 by reversing the inhibitory effect of IL-17A on SIRT1, which may be a potential mechanism by which glycyrrhizin improves psoriasis.

Psoriasis is a chronic immune skin disease mediated by T cells, keratinocytes, dendritic cells, and other immune cells. The interaction between IL-17A secreted by TH17 cells and epidermal keratinocytes plays a key pathogenic role in triggering psoriasis [16]. It has been reported that glycyrrhizin inhibits liver fibrosis by regulating the balance of Th1 / Th2 and TH17 / regulatory T cells in mouse models of liver fibrosis [17]. Moreover, glycyrrhizin combined with acitretin has been shown to inhibit the synergistic effects produced by TH17 cells, and improve psoriasis [3]. In this study, we found that glycyrrhizin can improve the adverse reactions and pathological status of IMD mice by reducing the serum expression of IL-17A, which suggests that glycyrrhizin may inhibit the secretion of IL-17A in TH17 cells and reduce the effects of IL-17A on other cells.

Increased levels of IL-17A will produce a self-amplifying inflammatory response in keratinocytes and promote keratinocyte proliferation, which will further promote the formation of mature psoriatic plaques [15]. In this study, the CCK-8 results indicated that glycyrrhizin substantially inhibited the proliferation of IL-17A-HaCaT cells. In addition to IL-17A, IFN-γ can also promote keratinocyte growth [16]. Using an ELISA assay, we found that glycyrrhizin not only inhibits IL-17A expression, but also inhibits IFN-γ expression. Therefore, glycyrrhizin might inhibit the growth of keratinocytes by inhibiting the expression of IL-17A and IFN-γ.

The expression of TNF-α, IL-6, and CCL20 is regulated by IL-17A [9, 10]. We found that in the lysate and supernatant of HaCaT cells, glycyrrhizin significantly reversed the promotion of TNF-α, IL-6, and CCL20 induced by IL-17A. In addition to IL-17A, STAT3 has been reported to regulate the expression of TNF-α, IL-6, and CCL20 [18]. Moreover, SIRT1 is involved in regulating the expression of STAT3 in various diseases, including cancer [19], diabetic kidneys [20] and hepatic gluconeogenesis [21]. In this study, we found that glycyrrhizin inhibited the expression of STAT3 by promoting the expression of SIRT1 in keratinocytes, which suggests that glycyrrhizin may also reduce the expression of TNF-α, IL-6, and CCL20 through the SIRT1-STAT3 pathway. IL-17A activates STAT3 in keratinocytes [22], and STAT3 also promotes the transcription of IL-17A [18]. Therefore, we speculate that glycyrrhizin reduces the expression of IL-17A by inhibiting the expression of STAT3, while the low expression of IL-17A further weakens the expression of STAT3 in keratinocytes, thereby forming a positive feedback pathway, which is beneficial for improving psoriasis.

Protein modifications not only affect protein homeostasis, but can also establish new cellular functions and play important and complex roles in cell signal transduction [23]. Protein phosphorylation is an important cellular regulatory mechanism in which enzymes and receptors are activated or inactivated by phosphorylation and dephosphorylation events via protein kinases [24]. In addition, acetylation of lysine residues is also a protein modification mechanism that regulates protein activity through the function of acetyltransferases [25]. STAT3 activation also depends on post-translational modifications, phosphorylation, and acetylation [26]. A previous report indicated that sunitinib reduces imiquimod-induced psoriasis-like inflammation by inhibiting p-STAT3 [27]. In this study, we found that glycyrrhizin can attenuate the promotion of p-STAT3 induced by SIRT1, which suggests that glycyrrhizin may be beneficial for improving psoriasis by inhibiting the expression of p-STAT3. Moreover, IL-22 has a pathogenic role in psoriasis, and IFN-γ can enhance the basic expression of a-STAT3, thereby weakening the response of keratinocytes to IL-22 [28]. In this study, we found that glycyrrhizin can attenuate the promotion of a-STAT3 induced by SIRT1, which suggests that glycyrrhizin may attenuate the response of keratinocytes to IL-22 by inhibiting the expression of a-STAT3, thereby playing a role in improving psoriasis.

AMPK, as an evolutionarily conserved serine/threonine kinase, is considered to be a key factor in maintaining cell energy homeostasis, and is essential in regulating metabolic-inflammation [29, 30]. GLP-1 partially inhibits the inflammatory signals of HaCaT cells by activating AMPK [31]. Study has shown that MTX partially restores the immunosuppressive function of Tregs by activating AMPK [32]. In this study, we found that glycyrrhizin promotes the expression of p-AMPK, suggesting that glycyrrhizin exerts an immunosuppressive effect in HaCaT cells.

In summary, glycyrrhizin may reduce the secretion of IL-17A through the SIRT1-STAT3-IL-17A pathway in TH17 cells and keratinocytes in vivo, thereby weakening the regulation of IL-17A in other cells and improving psoriasis. Particularly, glycyrrhizin improves psoriasis by inhibiting the secretion of IL-17A and IFN-γ in TH17 cells. Meanwhile, the proliferation of keratinocyte was reduced when Th17 cells secreted less IL-17A and IFN-γ, which also played a role in improving psoriasis. As for keratinocytes, glycyrrhizin may inhibit keratinocyte proliferation through the SIRT1-STAT3 pathway. In addition, through the SIRT1-dependent pathway, the inhibition of p-STAT3 and a-STAT3 expression by glycyrrhizin may also be related to the improvement of psoriasis. Together, the SIRT1-STAT3 pathway may be the key for glycyrrhizin to improve psoriasis, and this pathway may not be limited to TH17 cells and keratinocytes. Therefore, in future studies, we will elucidate whether glycyrrhizin can regulate additional cells related to psoriasis through the SIRT1-STAT3 pathway.

## Supplementary Information

**Additional file 1: Supplement Figure 1**. The experimental design in vivo

**Additional file 2: Supplement Figure 2**. Glycyrrhizin-mediated inflammation inhibition is due to its own cytotoxicity. (A) CCK-8; (B-D) ELISA. *P < 0.05, **P < 0.01 and ***P < 0.001; GA represents glycyrrhizin

## Data Availability

The datasets generated and/or analysed during the current study are not publicly available due to the commercial interests of Shenzhen Jianan (Group) Co., Ltd., but are available from the corresponding author on reasonable request.
