# Supplementary figures and images for: Glycyrrhizin improves the pathogenesis of psoriasis partially through IL-17A and the SIRT1-STAT3 axis
Source: BMC Immunol. 2021 May 27;22:34. doi: 10.1186/s12865-021-00421-z (PMC8161965; doi:10.1186/s12865-021-00421-z)

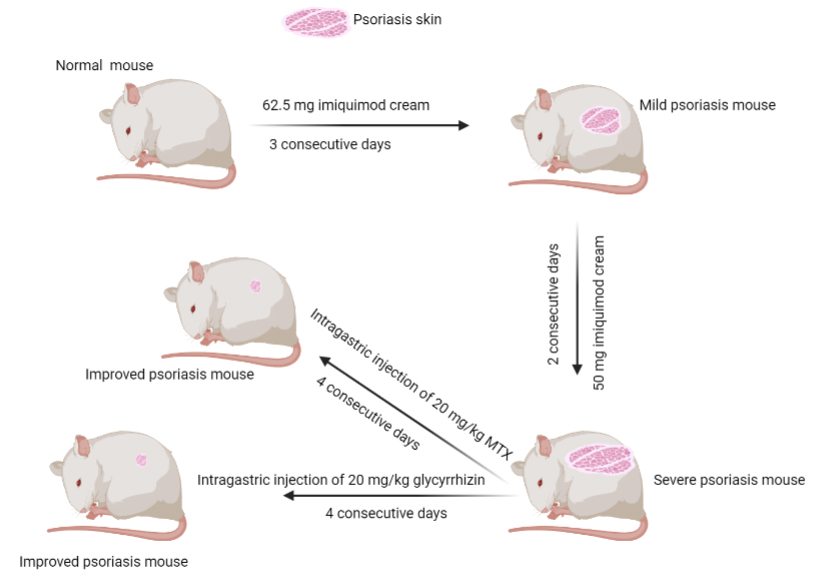

Supplement: Supplementary file 1 — Additional file 1: Supplement Figure 1. The experimental design in vivo [file 12865_2021_421_MOESM1_ESM.jpg]

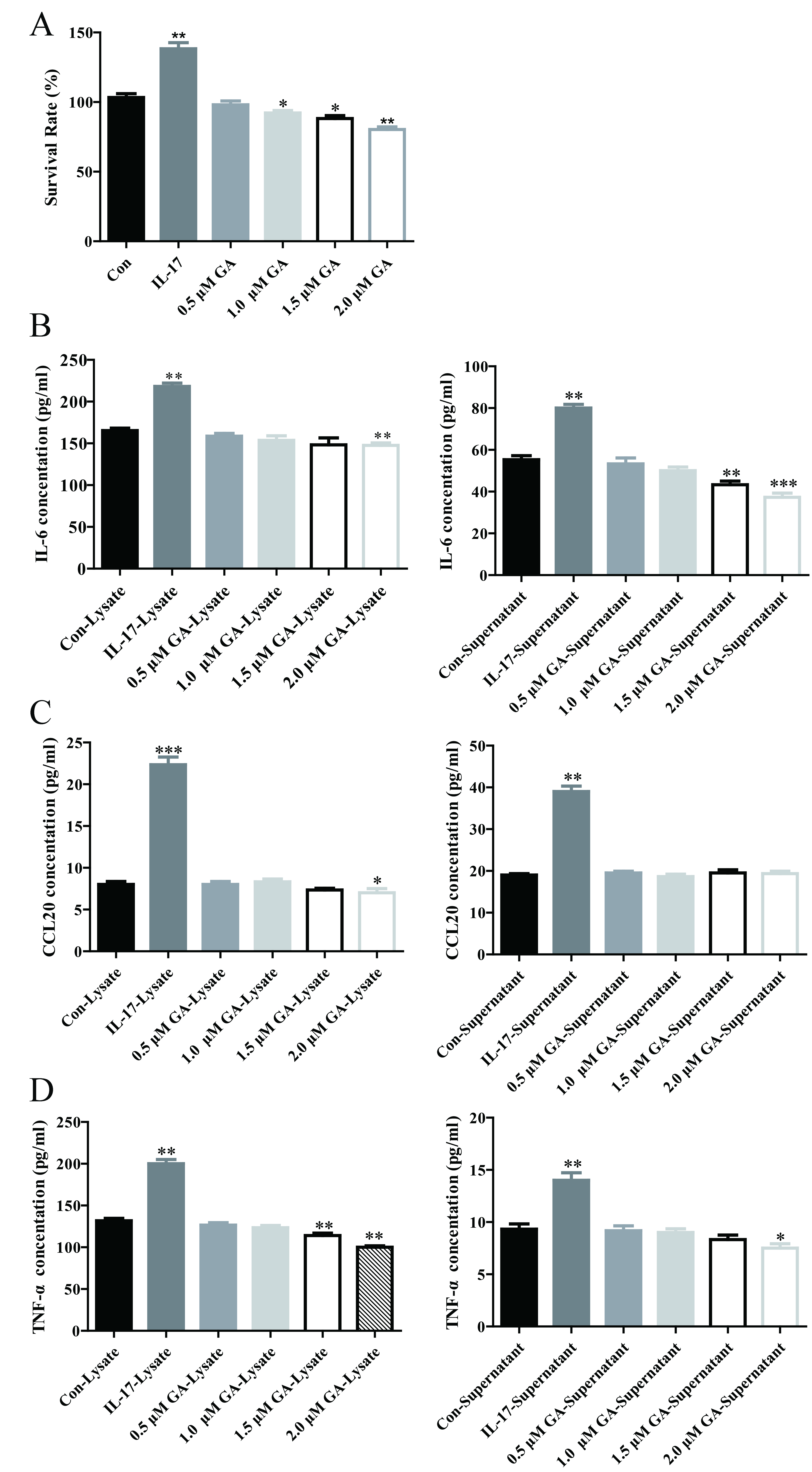

Supplement: Supplementary file 2 — Additional file 2: Supplement Figure 2. Glycyrrhizin-mediated inflammation inhibition is due to its own cytotoxicity. (A) CCK-8; (B-D) ELISA. *P < 0.05, **P < 0.01 and ***P < 0.001; GA represents glycyrrhizin [file 12865_2021_421_MOESM2_ESM.jpg]
